# Supplementary material for: Centrosomal Protein 55 (CEP55) Drives Immune Exclusion and Resistance to Immune Checkpoint Inhibitors in Colorectal Cancer
Source: Vaccines (Basel). 2024 Jan 8;12(1):63. doi: 10.3390/vaccines12010063 (PMC10820828; doi:10.3390/vaccines12010063)
Supplement: Supplementary file 1 [file vaccines-12-00063-s001.zip › vaccines-2746816-supplementary.pdf]

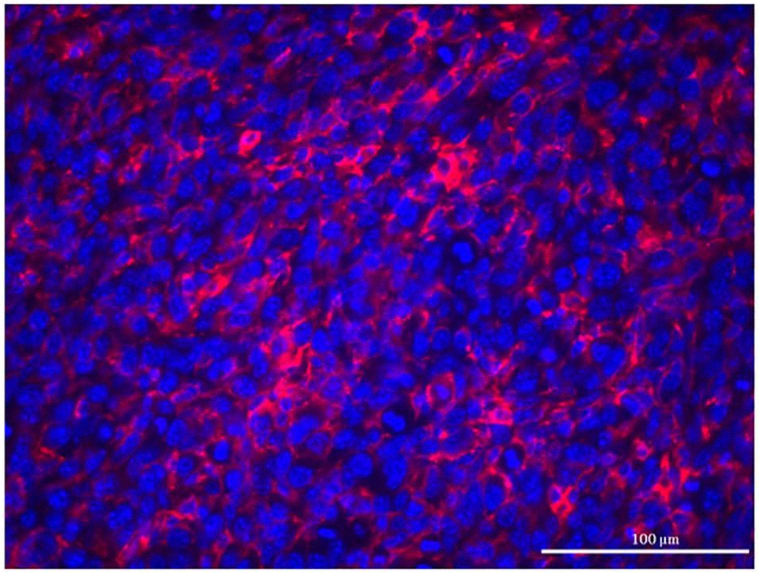

CT26 CEP55 KO

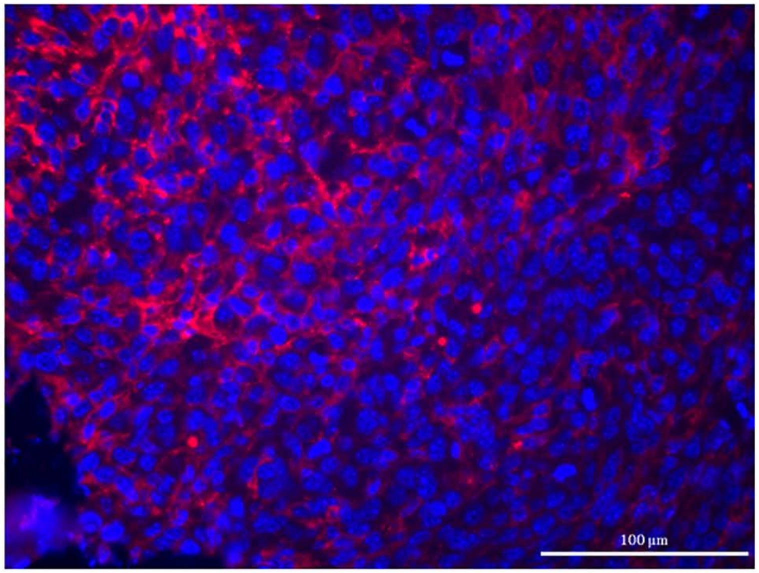

CT26 CEP55 WT

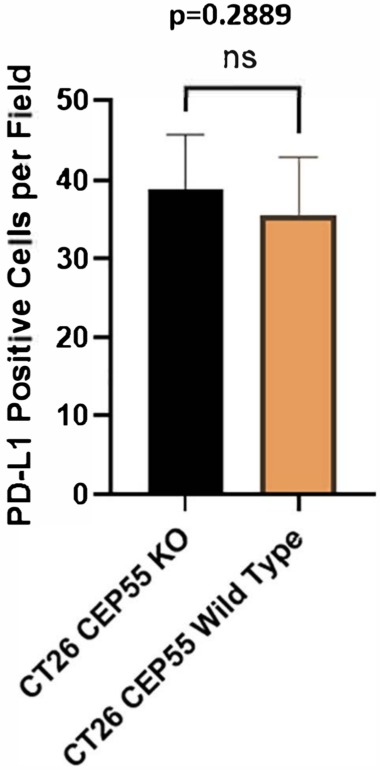

Supplemental Figure 1: Immunofluorescence of PD-L1 expression in CT26 CEP55 KO and CT26 CEP55 WT tumors treated with anti-PD1 therapy. There was no significant difference noted in PD-L1 expression between CT26 CEP55 KO and CT26 CEP55 WT tumors (p=0.2998).
